# Chromosomal Damage and Apoptosis in Exfoliated Buccal Cells from Individuals with Oral Cancer

**DOI:** 10.1155/2012/457054

**Published:** 2012-01-19

**Authors:** Lavínia Tércia Magalhães Dórea, José Roberto Cardoso Meireles, Júlia Paula Ramos Lessa, Márcio Campos Oliveira, Carlos Alberto de Bragança Pereira, Adriano Polpo de Campos, Eneida de Moraes Macílio Cerqueira

**Affiliations:** ^1^Laboratory of Toxicological Genetics, Department of Biological Science, Feira de Santana State University, Avendia Transnordestina s/n, 44.036-900 Feira de Santana, BA, Brazil; ^2^Buccal Pathology Laboratory, Department of Health, Feira de Santana State University, Avendia Transnordestina s/n, 44.036-900 Feira de Santana, BA, Brazil; ^3^Department of Mathematics and Statistics, São Paulo State University, Rua do Mãtao 1010, 05508-090 São Paulo, SP, Brazil; ^4^Department of Statistics, São Carlos Federal University, Via Washington Luis Km 235, 13565-905 São Carlos, SP, Brazil

## Abstract

This study aimed to investigate cytological abnormalities indicative of chromosome damage (micronuclei) and apoptosis (karyorrhexis, pyknosis, and condensed chromatin) in exfoliated cells from the buccal mucosa of patients with oral cancer and control subjects. The sample included twenty individuals with oral cancer and forty individuals with normal buccal mucosa. Material was collected from the cheek epithelium in areas with lesions and areas without abnormalities. A minimum of one thousand cells was analyzed. Micronuclei were found significantly more frequently in cells collected from lesions than in cells from normal areas, independent of the presence/absence of cancer (*P* < 0.0001). They were also significantly more frequent in smokers and in mouthwash users (*P* < 0.0001). Apoptosis occurred significantly less frequently in individuals with oral cancer (*P* < 0.0001). These results show that oral cancer is associated with higher frequency of chromosomal damage and suggest that apoptosis is compromised in the buccal cells of individuals with this kind of neoplasia.

## 1. Introduction

Oral cancer is among the ten types of malignant neoplasia of highest incidence worldwide and is particularly common in developing countries [1, 2]. Cigarette smoking is considered to be the most important risk factor for its development, particularly when in association with alcoholic beverages [3–5].

Similarl to other types of malignant neoplasia, oral cancer results from alterations (point mutations and chromosomal abnormalities) in genes that control the cell cycle, and/or in genes that are involved in DNA repair. In addition to the potential for metastasis, cancer is characterized by the loss of the ability of cells to evolve to death when genetic damage occurs (apoptosis) [6].

Occurrences of chromosomal damage in the oral epithelium can be evaluated using the micronucleus test, as suggested by Stich et al. [7]. Micronuclei are formed by chromosome fragments or whole chromosomes that fail to be included in the nuclei during cell division. They remain in the cytoplasm of interphase cells, where they can be observed as structures resembling nuclei [8]. The sensitivity of this test can be improved if, in addition to counting micronuclei, degenerative alterations indicative of apoptosis (karyorrhexis, pyknosis, and condensed chromatin) are also investigated [9, 10].

In the present study, chromosome damage and apoptosis were investigated in exfoliated cells from the buccal mucosa of patients with oral cancer and control subjects, using the protocols suggested by Tolbert et al. and Thomas et al. [9, 10]. Induction of micronuclei by means of risk factors for oral cancer was also evaluated.

## 2. Methods

### 2.1. Sample Collection and Preparation

Exfoliated cells from the buccal mucosa were obtained from twenty patients with oral cancer (case group) and forty individuals without oral lesions (control group). The individuals in both groups were attended by the dentistry services of Feira de Santana State University. Clinical examinations of oral cavity were performed on all individuals in the sample. Biopsies were performed by the dentist, and histopathological diagnoses were made by a pathologist within a specific service at this University. The sample was characterized using a questionnaire that asked about risk factors for oral cancer development: cigarette smoking, alcoholic beverage ingestion, oral hygiene, and mouthwash use. Individuals who, for at least one year, had been consumed three or more cigarettes/day were considered to be smokers [11]. Individuals who said that they consumed alcoholic beverages two or three times a week were considered to be drinkers [12]. The material for analysis was collected from the cheek mucosa in areas without lesions, from individuals in both the case group and the control group, and in areas with lesions from individuals in the case group, by means of gentle scraping of the epithelium using a cytobrush. From the material collected, smears were prepared on clean slides onto which two drops of saline solution (0.9% NaCl) had previously been placed. After air drying, the slides were fixed in a methanol/acetic acid solution (3 : 1) and, 24 hours later, were stained using the Schiff reagent and counterstained using 1% fast green.

### 2.2. Cytological Analysis

**Figure 1:**
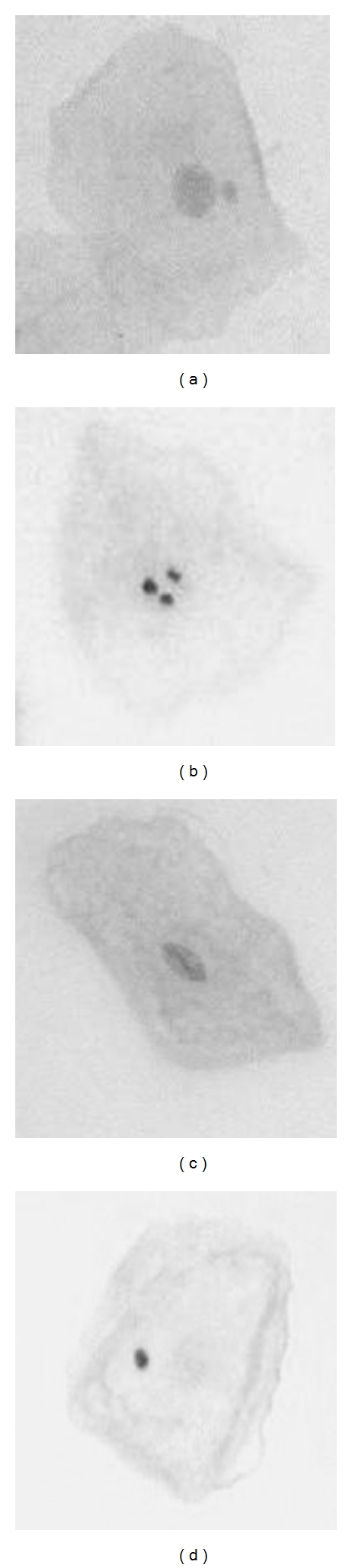
Cells presenting a micronucleus (a) karyorrhexis (b), condensed chromatin (c), and pyknosis (d).

The slides were analyzed under an optical microscope in a blinded manner. A minimum of 1,000 cells presenting intact cytoplasm were counted. The analysis protocol used was as suggested by Tolbert el al. and Thomas et al. [9, 10]. In according with these protocols, in addition to counting micronuclei, nuclear alterations suggestive of apoptosis were also investigated: karyorrhexis, condensed chromatin, and pyknosis (Figure 1). The criteria adopted for identifying of these structures were those described by Sarto et al. [13] and Tolbert et al. and Thomas et al. [9, 10].

### 2.3. Statistical Analysis

Differences between the mean ages of the groups were evaluated using Student's *t*-test. The chi-square and Fisher tests were used to analyze association tables. Differences in micronucleus presence and nuclear degenerative alterations relating to apoptosis occurrence were evaluated using the conditional test for evaluation of proportions in situations of rare events, as suggested by Bragança-Pereira [14]. Beside these marginal analyses for each of the end points, we performed a logistic regression analysis [15] to consider at the same time the influence of all the end points. We have performed two logistic regression analyses. The first uses cells from health tissues in the control group and cells from tumor tissues in the case group. The second uses only cells from health tissues in both groups. The significance level used for all the analyses was 5%.

### 2.4. Ethical Matters

In accordance with Resolution number 196/1996 of the Brazilian National Health Board, all the participants signed an informed consent statement and full confidentiality was ensured. The study was approved by the Ethics Committee of Feira de Santana State University (Protocol number 059/2006).

## 3. Results

### 3.1. Sample Characteristics

The mean age ±SE of the whole sample was 55.53 ± 2.06. For the case and control groups, respectively, the means were 63.25 ± 3.49 and 51.68 ± 2.34. Student's *t*-test indicated that there was a significant difference between the groups (*P* = 0.007). The groups did not differ in relation to gender (*P* = 0.094), buccal hygiene (*P* = 0.493), use of oral antiseptics (*P* = 0.221), or tobacco consumption (*P* = 0.064). All the individuals who said that they were drinkers were also smokers. The number of drinkers in the case group was significantly higher than in the control group (*P* = 0.002). These data are shown in Table 1.

### 3.2. Statistical Analysis Using the Conditional Test for Evaluation of Proportions in Situations of Rare Events

#### 3.2.1. Micronucleus Analysis

Micronucleus occurrence was significantly higher in cells obtained from areas with lesions in the case group than in cells obtained from areas without lesions in both the case group and the control group (*P* < 0.0001). No difference was observed in comparing cells obtained from the control group and from normal areas in the case group (*P* = 0.7964). These data are presented in Table 2.

In comparing cells obtained from normal areas of the groups, no difference in micronucleus occurrence was observed in relation to age, gender, or oral hygiene. However, micronucleus occurrence was significantly higher in mouthwash users (*χ* ^2^ = 21.4224; DF = 1; *P* < 0.0001). The effects of cigarette smoking and alcohol consumption were evaluated also considering cells obtained from normal areas of the groups, with the sample divided into three subgroups: (A) smokers; (B) nonsmokers and nondrinkers; (C) smokers and drinkers (Table 3).

#### 3.2.2. Apoptosis Analysis

Data relative to degenerative nuclear alterations indicative of apoptosis are presented in Table 4.

As observed in Table 5 apoptosis (Σ karyorrhexis, condensed chromatin, and pyknosis) occurred significantly less frequently in cells obtained from lesion areas than in cells from the control group (*P* < 0.0001). It was also less frequent in cells from normal areas in the case group than in normal areas in the control group (*P* < 0.0001). There was no difference in apoptosis occurrence between cells from lesion areas and cells from normal areas in the case group (*P* = 0.4786).

### 3.3. Statistical Analysis using a Logistic Regression

The first comparison (using cells from health tissues in the control group and cells from tumor tissues in the case group) shows that MN is the most important end point and age should be disregarded. The second comparison (using only cells from health tissues in both groups) shows that age becomes important and MN can be disregarded. These results are much sounded since the first comparison involves cells from tumor tissues and the second only cells from health tissues. After the models adjustment and the elimination of nonsignificant end points, we obtain the following models:

- comparing cells from tumor tissues in the case group with health tissues in the control group the logistic regression function is as follows:
  (1) $$\;\begin{array}{cc} & P\left(\text{belong}\;\text{case}\;\text{group}\;|\;\text{data}\right)\\ & \;\;=\{1+\exp[-(-1.588\text{DS}-.111\text{carx}\;\\ & {\;\;\;\;\;\;\;\;+.374\text{pic}+1.537\text{MN})]\}}^{-1}, \end{array}$$
- comparing cells from health tissues in both groups, case and control, the logistic regression function change to the following:
  (2) $$\begin{array}{cc} & P\left(\text{belong}\;\text{case}\;\text{group}\;|\;\text{data}\right)\\ & \;\;=\{1+\exp[-(-2.416\text{DS}-.086\text{carx}\;\\ & {\;\;\;\;\;\;\;\;+.332\text{pic}+.047\text{age})]\}}^{-1}. \end{array}$$

Finally, calculating the values of these functions for all the sample unities we made use of the ROC curve to define cut-off values and then evaluate the sensibility and specificity of each of the two kinds of comparisons. The result was impressive since the sensibility for both comparisons were 80% and the specificity change from 95% in the first comparison to 85% in the second. The area under the ROC curve changes from  .9462 for the first model to  .8762 for the second model. This proves the good fit of both kinds of model to the data analyzed.

## 4. Discussion

Occurrences of chromosome damage and their association with cancer development have been evaluated using the micronucleus assay in both lymphocytes and exfoliated cells from some types of epithelium [11, 16–18]. In the oral epithelium, micronuclei are considered to be important biomarkers for the risk of cancer development [16, 18–21].

The higher frequency of micronuclei in exfoliated cells from malignant lesions observed in this study corroborates the results described by several other authors [16, 18–21], thereby indicating the usefulness of micronuclei as biomarkers for the risk of cancer in the oral epithelium. In the same way as described by Casartelli et al. [16], micronucleus occurrences did not differ in cells obtained from normal mucosa, between individuals with and without oral cancer.

In agreement with some results previously described [22, 23], no association between age and micronucleus occurrence was observed in the present study. However, other authors have shown such an association [24–26]. In addition, the present study did not find any association between micronucleus occurrence and gender, concordant with the results from some studies [23, 27, 28], although this association has been described by others [29–31]. Like Bloching et al. [26], the present study did not find any association between micronucleus occurrence and oral hygiene.

The greater occurrence of micronuclei in mouthwash users was also observed in a study that evaluated the genotoxic effects of risk factors for oral cancer development [32]. However, this association must be viewed with caution, since seven of those users were also smokers.

Induction of micronuclei in exfoliated buccal cells consequent to smoking has generated controversy in the literature. It has been suggested that this association is dependent on the number of cigarettes consumed, since it was observed only among users of more than ten cigarettes/day [25, 26]. The smokers analyzed in the present study also had this level of consumption. Synergistic effects between smoking and drinking have been described [33–35], but no such effects were observed in the present study.

The lower frequency of apoptosis observed in both the lesion and the normal areas in the case group indicate that, with evolution of malignant transformation, the apoptotic response fails, as also observed in precursor lesions of cervical cancer [36].

## 5. Conclusions

The results obtained in the present study show that oral cancer is associated with a higher frequency of chromosome damage and suggest that apoptosis is impaired in the buccal cells of individuals with this kind of neoplasia. Additionally, they suggest that tobacco and mouthwashes are effective in inducing chromosome damage. The inclusion of degenerative nuclear alteration indicative of apoptosis beside micronucleus is useful to biomonitoring oral cancer.

## Figures and Tables

**Table 1:** Sample characteristics.

Characteristic	Group				*P* value
	Case		Control		
	*N* = 20		*N* = 40		
	*N*	%	*N*	%	
Gender					
Female	9	45.0	27	67.5	0.094^b^
Male	11	55.0	13	32.5	
Tobacco consumption					
Yes	15	75.0	20	50.0	0.064^b^
No	5	25.0	20	50.0	
Drinker					
Yes	8	40.0	3	7.5	0.002^a^
No	12	60.0	37	92.5	
Tobacco consumption and drinker					
Yes	8	53.3	3	15.0	0.016^a^
No	7	46.7	17	85.0	
Oral hygiene					
Good	1	5.0	5	10.3	0.493^b^
Poor	19	95.0	35	89.7	
Mouthwash use					
Yes	5	25.0	5	12.5	0.221^b^
No	15	75.0	35	87.5	

^a^Significant; ^b^nonsignificant.

**Table 2:** Micronucleus (MN) analysis.

Group	*N*	MN (n°)	MN (‰)	Total cells	Comparison	*χ* ^2^ (DF = 1)
			Mean ± SE			
Case^L.A^	20	76	2.07 ± 0.39	41,079	Case^L.A^ versus Control	60.9647; *P* < 0.0001^a^
Case^N.A^	20	25	0.61 ± 0.25	51,153	Case^L.A^ versus Case^N.A^	38.5582; *P* < 0.0001^a^
Control	40	41	0.42 ± 0.14	89, 568	Case^N.A^ versus Control	0.0666; *P* = 0.7964^b^

^L.A^Lesion area, ^N.A^normal area, ^a^significant, ^b^nonsignificant.

**Table 3:** Data relating to micronucleus occurrence in smokers (A), nonsmokers and nondrinkers (B), and smokers and drinkers (C).

Subgroup	*N*	Micronucleus	Total cells	*χ* ^2^	*χ* ^2^ partitions (DF = 1)
A	24	35	61,983	8.4734	A versus B: *χ* ^2^ = 6.0345; *P* = 0.0140^a^
B	25	15	55,734	df = 2	A versus C: *χ* ^2^ = 0.4788; *P* = 0.4890^b^
C	11	16	23,004	*P* = 0.0145	B versus C: *χ* ^2^ = 7.5195; *P* = 0.0061^a^

Total	60	66	140,721		

^a^Significant, ^b^nonsignificant.

**Table 4:** Degenerative nuclear alterations indicative of apoptosis observed.

Group	*N*	Total cells	Karyorrhexis	Condensed chromatin	Pyknosis
Case^L.A^	20	41,079	334	592	175
Case^N.A^	20	51,153	393	803	136
Control	40	89,568	1,803	3,349	77

^L.A^Lesion area, ^N.A^normal area, ^a^significant, ^b^nonsignificant.

**Table 5:** Apoptosis analysis (Σ karyorrhexis, condensed chromatin and pyknosis).

Group	*N*	Apoptosis (n°)	Apoptosis (‰)	Total cells	Comparison	*χ* ^2^; *P* (DF = 1)
			Mean ± SE			
Case^L.A^	20	1,101	27.81 ± 3.45	41,079	Case^L.A^ versus Control	579.62; <0.0001^a^
Case^N.A^	20	1,332	31.49 ± 6.60	51,153	Case^L.A^ versus Case^N.A^	0.5021; = 0.4786^b^
Control	40	5,229	58.08 ± 12.65	89,568	Case^N.A^ versus Control	730.39; <0.0001^a^

^L.A^Lesion area, ^N.A^normal area, ^a^significant, ^b^nonsignificant.

## References

[1] Marchioni DML, Fisberg RMJ, De Góis Filho JF (2007). Fatores dietéticos e câncer oral: estudo caso-controle na Região Metropolitana de São Paulo, Brasil. *Cadernos de Saúde Pública*.

[2] Warnakulasuriya S (2009). Global epidemiology of oral and oropharyngeal cancer. *Oral Oncology*.

[3] Wünsch-Filho V (2002). The epidemiology of oral and pharynx cancer in Brazil. *Oral Oncology*.

[4] Varela-Lema L, Ruano-Ravina A, Juiz Crespo MAJ, Barros-Dios JM (2010). Tobacco consumption and oral and pharyngeal cancer in a Spanish male population. *Cancer Letters*.

[5] Rodriguez T, Altieri A, Chatenoud L (2004). Risk factors for oral and pharyngeal cancer in young adults. *Oral Oncology*.

[6] Hanahan D, Weinberg RA (2000). The hallmarks of cancer. *Cell*.

[7] Stich HF, Curtis JR, Parida BB (1982). Application of the micronucleus test to exfoliated cells of high cancer risk groups: tobacco chewers. *International Journal of Cancer*.

[8] Holland N, Bolognesi C, Kirsch-Volders M (2008). The micronucleus assay in human buccal cells as a tool for biomonitoring DNA damage: the HUMN project perspective on current status and knowledge gaps. *Mutation Research*.

[9] Tolbert PE, Shy CM, Allen JW (1992). Micronuclei and other nuclear anomalies in buccal smears: methods development. *Mutation Research*.

[10] Thomas P, Holland N, Bolognesi C (2009). Buccal micronucleus cytome assay. *Nature Protocols*.

[11] Cerqueira EMM, Santoro CL, Donozo NF (1998). Genetic damage in exfoliated cells of the uterine cervix: association and interaction between cigarette smoking and progression to malignant transformation?. *Acta Cytologica*.

[12] Kehdy FSG, Cerqueira EMM, Bonjardim MB, Camelo RM, Castro MCL (2007). Study of the cytogenetic effects of occupational exposure to pesticides on sanitation workers in Belo Horizonte, Brazil. *Genetics and Molecular Research*.

[13] Sarto F, Finotto S, Giacomelli L (1987). The micronucleus assay in exfoliated cells of the human buccal mucosa. *Mutagenesis*.

[14] Bragança-Pereira CA, Rabello-Gay MN, Rodrigues MA, La R, Monteleone-Neto R (1991). Teste estatístico para comparar proporções em problemas de citogenética. *Mutagênese, Carcinogênese e Teratogênese: Métodos e Critérios de Avaliação*.

[15] Hosmer D, Lemeshow S (1989). *Applied Logistic Regression*.

[16] Casartelli G, Bonatti S, De FerrariM (2000). Micronucleus frequencies in exfoliated buccal cells in normal mucosa, precancerous lesions and squamous cellcarcinoma. *Analytical & Quantitative Cytology & Histology*.

[17] Leal-Garza CH, Cerda-Flores RM, Leal-Elizondo E, Cortés-Gutiérrez EI (2002). Micronuclei in cervical smears and peripheral blood lymphocytes from women with and without cervical uterine cancer. *Mutation Research*.

[18] Halder A, Chakraborty T, Mandal K (2004). Comparative study of exfoliated oral mucosal cell micronuclei frequency in normal, precancerous and malignant epithelium. *International Journal of Human Genetics*.

[19] Kamboj M, Mahajan S (2007). Micronucleus—an upcoming marker of genotoxic damage. *Clinical Oral Investigations*.

[20] Saran R, Tiwari RK, Reddy PP (2008). Risk assessment of oral cancer in patients with pre-cancerous states of the oral cavity using micronucleus test and challenge assay. *Oral Oncology*.

[21] Chatterjee S, Dhar S, Sengupta B (2009). Cytogenetic monitoring in human oral cancers and other oral pathology: the micronucleus test in exfoliated buccal cells. *Toxicology Mechanisms and Methods*.

[22] Karahalil B, Karakaya AE, Burgaz S (1999). The micronucleus assay in exfoliated buccal cells: aplication to occupational exposure to polycyclic aromatic hydrocarbons. *Mutation Research*.

[23] Konopacka M (2003). Effect of smoking and aging on micronucleus frequencies in human exfoliated buccal cells. *Neoplasma*.

[24] Pinto D, Ceballos JM, García G (2000). Increased cytogenetic damage in outdoor painters. *Mutation Research*.

[25] Wu PA, Loh CH, Hsieh LL, Liu TY, Chen CJ, Liou SH (2004). Clastogenic effect for cigarette smoking but not areca quid chewing as measured by micronuclei in exfoliated buccal mucosal cells. *Mutation Research*.

[26] Bloching M, Reich W, Schubert J, Grummt T, Sandner A (2008). Micronucleus rate of buccal mucosal epithelial cells in relation to oral hygiene and dental factors. *Oral Oncology*.

[27] Maffei F, Angelini S, Forti GC (2002). Micronuclei frequencies in hospital workers occupationally exposed to low levels of ionizing radiation: Influence of smoking status and other factors. *Mutagenesis*.

[28] Joseph LJ, Patwardhan UN, Samuel AM (2004). Frequency of micronuclei in peripheral blood lymphocytes from subjects occupationally exposed to low levels of ionizing radiation. *Mutation Research*.

[29] Fenech M (2001). The role of folic acid and Vitamin B12 in genomic stability of human cells. *Mutation Research*.

[30] Jagetia GC, Jayakrishnan A, Fernandes D, Vidyasagar MS (2001). Evaluation of micronuclei frequency in the cultured peripheral blood lymphocytes of cancer patients before and after radiation treatment. *Mutation Research*.

[31] Zakeri F, Assaei RG (2004). Cytogenetic monitoring of personnel working in angiocardiography laboratories in Iran hospitals. *Mutation Research*.

[32] Freita VS, Lopes MA, Meireles JRC (2005). Genotoxic effects of factors considered of risk for the buccal cancer. *Revista Baiana de Saúde Pública*.

[33] Blot WJ, McLaughlin JK, Winn DM (1988). Smoking and drinking in relation to oral and pharyngeal cancer. *Cancer Research*.

[34] Castellsagué X, Quintana MJ, Martínez MC (2004). The role of type of tobacco and type of alcoholic beverage in oral carcinogenesis. *International Journal of Cancer*.

[35] Rodriguez T, Altieri A, Chatenoud L (2004). Risk factors for oral and pharyngeal cancer in young adults. *Oral Oncology*.

[36] Rogovskaya SI, Sukhikh GT, Zhdanov AV, Kolobova EA, Ezhova LS (2001). Apoptosis in woman uterine cervix in pathologies associated with human papillomavirus. *Bulletin of Experimental Biology and Medicine*.

